# A comparative dosimetric study of hypofractionated radiotherapy with different target volume delineation approaches in breast cancer patients after implant-based reconstruction

**DOI:** 10.3389/fonc.2025.1552813

**Published:** 2026-01-06

**Authors:** Lipeng Ding, Xu Wang, Zhangcai Zheng, Jianping Long, Guoying Miao, Tao Yang, Shuxia Li, Na Dong, Liying Gao

**Affiliations:** 1 Department of Radiation Oncology, Gansu Provincial Maternity and Child Health Hospital, Lanzhou, China; 2 The First School of Clinical Medicine, Lanzhou University, Lanzhou, China

**Keywords:** breast cancer, ESTRO-ACROP, immediate breast reconstruction, implants, radiotherapy, VMAT

## Abstract

**Purpose:**

This study dosimetrically compares hypofractionated VMAT plans using the updated ESTRO-ACROP guidelines versus conventional delineation in patients undergoing immediate implant-based breast reconstruction after mastectomy.

**Methods:**

We retrospectively enrolled 22 patients with immediate implant-based reconstruction post-mastectomy (12 left-sided, 10 right-sided), treated between January 2022 and June 2025. All patients underwent CT simulation; those with left-sided cancer were positioned using deep inspiration breath-hold (DIBH), and those with right-sided cancer under free breathing. For each patient, conventional (C-TVD) and ESTRO-ACROP guideline-based (E-TVD) target volumes were independently delineated on the same CT dataset. Hypofractionated VMAT plans were designed using a 6-MV beam, single-isocenter, dual-arc technique, prescribing 40.05 Gy in 15 fractions to the planning target volume (PTV). All plans were normalized to ensure ≥95% PTV coverage by the prescribed dose. Dose-volume parameters for targets and organs at risk (OARs) were then compared between the two delineation approaches.

**Results:**

The conformity index (CI) of E-TVD was inferior to that of C-TVD; however, E-TVD achieved superior 95% prescription dose coverage of the target volume. Compared with C-TVD, E-TVD resulted in significantly lower V_20_ and D_mean_ to the ipsilateral lung, with differences reaching statistical significance (P < 0.05). For the heart, E-TVD was associated with significantly lower V_20_, as well as lower D_max_ and D_mean_ to the left anterior descending coronary artery (LAD), with all differences reaching statistical significance (P < 0.05). Subgroup analyses stratified by left versus right breast cancer revealed that in left breast cancer patients, E-TVD resulted in statistically significant reductions in ipsilateral lung V_20_, V_10_, and D_mean_; bilateral lung V_20_; heart V_20_; LAD D_max_ and D_mean_; and contralateral breast D_mean_ (all P < 0.05). In right breast cancer patients, E-TVD was associated with significantly lower ipsilateral lung D_mean_ and contralateral breast D_mean_ (both P < 0.05).

**Conclusions:**

In patients with breast cancer who undergo total mastectomy followed by immediate implant-based breast reconstruction, the E-TVD approach confers superior protection to organs at risk.

## 1 Introduction

Total mastectomy remains an indispensable therapeutic strategy for patients with locally advanced or high-risk breast cancer (1). In recent years, the widespread adoption of implant-based breast reconstruction has markedly enhanced patients’ quality of life and psychosocial outcomes (2, 3). The indications for postoperative radiotherapy in patients undergoing breast reconstruction are identical to those in non-reconstructed patients; however, the presence of implants introduces unique considerations in target volume delineation. To optimize normal tissue sparing and mitigate reconstruction-related complications, the 2019 updated consensus statement issued by the European Society for Radiotherapy and Oncology and the Advisory Committee for Radiation Oncology Practice (ESTRO-ACROP) explicitly recommends excluding the implant itself during target volume delineation (4). This fundamental difference significantly alters the pattern of radiotherapy dose distribution. Currently, systematic comparative evidence regarding dosimetric parameters between these two delineation approaches remains relatively scarce, particularly in the context of hypofractionated radiotherapy.

Hypofractionated radiotherapy not only demonstrates comparable efficacy to conventional fractionated radiotherapy but also offers advantages such as reducing the number of treatment fractions, alleviating patients’ medical economic burden, and shortening hospital stay duration (5–7). Postmastectomy radiation therapy (PMRT) may elevate the risk of breast-related complications following breast reconstruction, including wound infection, dehiscence, capsular contracture, and implant rupture—complications that can result in poor aesthetic outcomes or even the need for revision surgery (8–10). By contrast, hypofractionated radiotherapy has been associated with lower rates of both breast complications and treatment interruptions in patients undergoing breast reconstruction (11). Consequently, hypofractionated regimens have emerged as the preferred approach for PMRT.

Against this backdrop, the present study hypothesizes that the target volume delineation approach recommended by the ESTRO ACROP guidelines confers dosimetric advantages over conventional target delineation methods in the context of hypofractionated radiotherapy. This hypothesis will be validated through comparative analysis of radiotherapy plans developed under these distinct delineation strategies.

## 2 Materials and methods

### 2.1 Patient information

Patients who underwent immediate implant-based breast reconstruction following total mastectomy for breast cancer at our institution between January 2022 and June 2025 were enrolled. The implants used included prostheses and tissue expanders. A total of 22 patients were enrolled, including 12 with left-sided breast cancer and 10 with right-sided breast cancer. The ages of the patients ranged from 27 to 59 years, with a mean age of 43.6 ± 6.7 years. According to the AJCC 8th edition staging system, tumor stages ranged from T1-3N1-2M0.

#### 2.1.1 Inclusion criteria

1. Pathologically confirmed malignant breast neoplasm, with total mastectomy and immediate implant-based breast reconstruction performed.
2. Presence of indications for postoperative radiotherapy.
3. Karnofsky Performance Status (KPS) score > 80.
4. Provision of written informed consent.

#### 2.1.2 Exclusion criteria

1. Presence of contraindications to radiotherapy.
2. Patients with unexpanded tissue expanders.

### 2.2 Pre-radiotherapy simulation localization techniques and scanning parameters

For simulation-based localization, a Brilliance large-bore CT scanner (Philips Healthcare, Netherlands) was utilized. Patient positioning was immobilized using a breast immobilization bracket or vacuum bag. The scanning range extended from the lower margin of the mandible (superior boundary) to the lower margin of the liver (inferior boundary), with a slice thickness of 3 mm. For patients with right-sided breast cancer, free-breathing localization was implemented. For those with left-sided breast cancer, DIBH localization was performed after they completed breathing training and achieved the required proficiency (parameter settings: inspiration volume of 1 L, breath-holding duration of 20–30 seconds).

### 2.3 Target volume delineation

All patients undergoing immediate implant-based breast reconstruction at our institution received subpectoral implant placement. For the updated target volume delineation, the method recommended by the ESTRO ACROP guidelines for subpectoral implant target volume delineation was adopted: If the dorsal fascia of the breast is not involved by cancer, the CTVp_chest wall for PMRT does not include the deep lymphatic plexus and therefore only includes the rim of tissue ventral to the major pectoral muscle and the implant, except at the medial, lateral and caudal borders where it may extend to the ventral side of the chest wall where it is not covered by the pre-surgical extension of the major pectoral muscle. Thus, the implant can be largely excluded from the CTVp_chest wall, whilst the parts of the chest wall surrounding the pectoral muscle around which the lymphatics flow should still be included. No risk factors (e.g., tumor involvement of the dorsal fascia) were identified in the enrolled patients; therefore, the tissue between the chest wall and the caudal end of the implant was not delineated (4).

**Figure 1:**
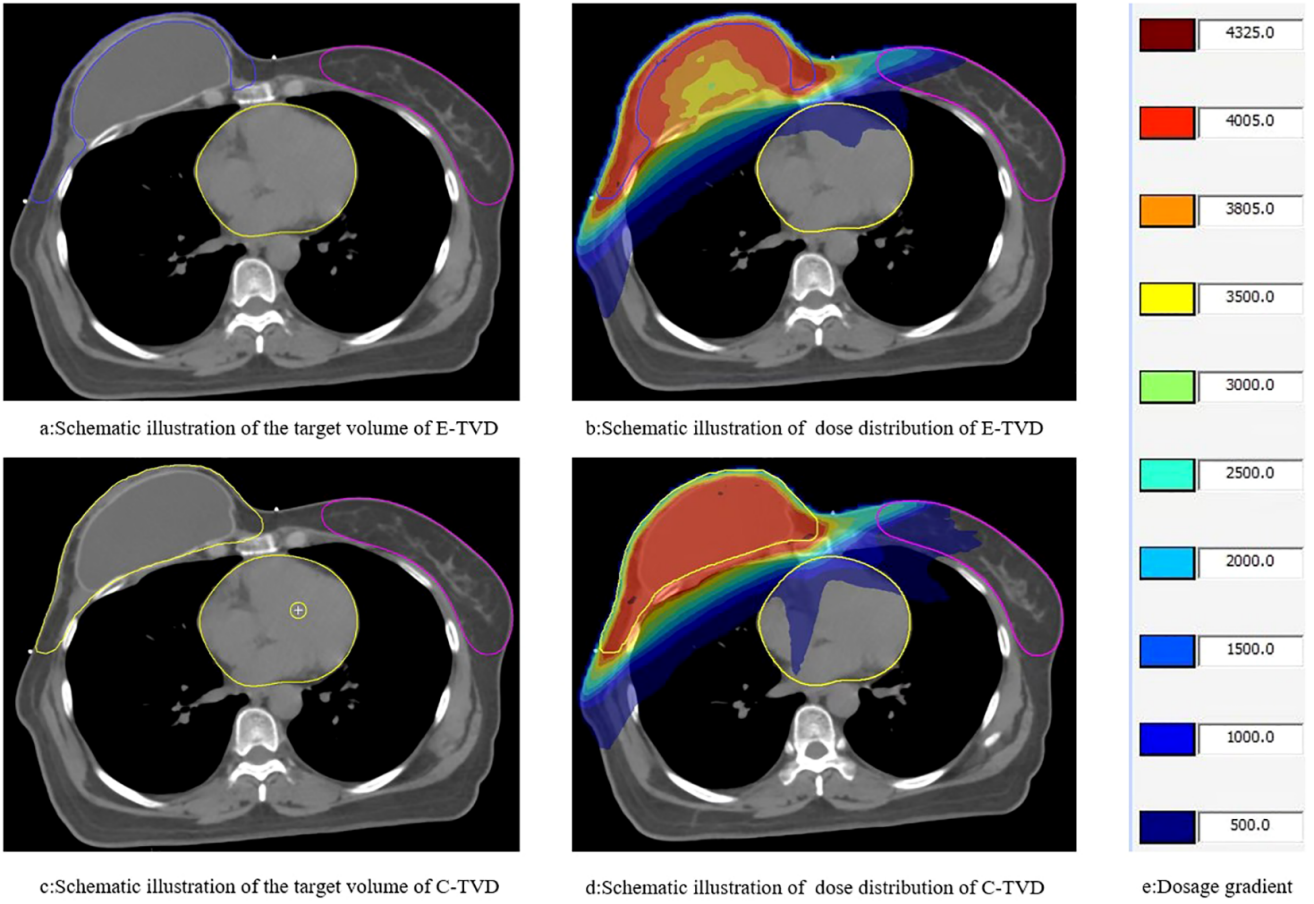
Schematic representation of the target area range and dose distribution in this study. **(a, b)** schematic illustration of the target area and dose distribution of E-TVD; **(c, d)** schematic illustration of the target area and dose distribution of C-TVD.

The conventional target volume (C-TVD) included the implant and was delineated following the ESTRO consensus guidelines for early-stage breast cancer (12), which were commonly used in our clinic prior to the adoption of the specific reconstruction guidelines. The PTV was generated by uniformly expanding the clinical target volume CTV by 5 mm, with a subsequent 3 mm retraction applied at the skin surface.For the delineation of organs at risk, the thyroid, both lungs, spinal cord, heart, LAD, contralateral breast, and humeral head on the affected side were delineated according to the RTOG atlas (13). We specified that the target volume be uniformly defined as the supraclavicular lymphatic drainage area + the axillary group III lymphatic drainage area + the redelineated chest wall (**Figure 1**).

To ensure the accuracy and reproducibility of target volume delineation, all target delineations were independently performed by two radiation oncologists with ≥5 years of experience in breast cancer radiotherapy target volume delineation. For each patient, the C-TVD and E-TVD were independently delineated by the two aforementioned physicians. Upon completion, cross-verification was performed. In cases of significant discrepancy (boundary deviation > 5 mm), a third senior radiation oncologist was consulted to arbitrate and finalize the contour.

### 2.4 Prescription and dose constraints for organs at risk (referring to the breast cancer radiation therapy guidelines, Chinese medical doctor association 2020 edition)

For radiation treatment, a 6-MV X-ray beam was used to administer 40.05 Gy to the planning target volume (PTV) in 15 fractions of 2.67 Gy each. Requirements: PTV V40.05 ≥95%, V38.05≥99%, V43.25 ≤5%. Heart: for left breast cancer: Dmean <8 Gy, V5 <40%; for right breast cancer: Dmean <5 Gy, V5 <30%. Ipsilateral lung: Dmean <15 Gy, V20 <30%, V5 <50%. Contralateral lung: V5 <20%. LAD: Dmean <25 Gy. Contralateral breast: Dmean <5–8 Gy. Thyroid gland: Dmean <30 Gy. Ipsilateral humeral head: V30 <20%. Liver: V5 <20%.(Note: Dmean represents the mean dose; Dmax represents the maximum dose; and Vx indicates the volume percentage of normal tissue receiving an x Gy radiation dose.).

### 2.5 Plan design and plan observation indicators

This study utilized the Elekta planning system and the Monte Carlo algorithm. The volumetric modulated arc therapy (VMAT) technique was employed with one field and two arcs, with arc angles ranging from 220° to 280°. Once the plan was completed, the prescription dose coverage of the PTV was normalized to 95% (**Figure 1**). The following dosimetric parameters were evaluated and compared between the two delineation methods: Target parameters (left breast, right breast): CTV_CW (chest wall CTV) volume, V108%, V95%, Dmax, monitor units (MU), conformity index (CI), and homogeneity index (HI). V108% represents the volume occupied by 108% of the prescription dose, while V95% represents the volume occupied by 95%. Organ at-risk doses (left breast, right breast): Ipsilateral lung: V20, V10, V5, and Dmean. Contralateral lung: V5, Dmean. Bilateral lungs: V20, V10, V5, and Dmean. Heart: V40, V30, V20, V10, V5, and Dmean. LAD: Dmax, Dmean. Contralateral breast: Dmean. Thyroid gland: Dmean. Liver: V5. (Note: Vx% refers to the volume encompassed by the xGy dose curve).

### 2.6 Statistical analysis

The Shapiro-Wilk test was employed to assess the normality of continuous variables, with the significance level set at α = 0.05. Data were considered to follow a normal distribution if P > 0.05. Measurement data satisfying normality were presented as $\bar{\text{x}}\pm \text{s}$ and compared using the paired t-test; those deviating from normality were reported as M (IQR) (via the quartile method) and analyzed using the Wilcoxon rank-sum test. A two-tailed P < 0.05 was deemed statistically significant. All data analyses were performed using SPSS 25.0 software.

## 3 Results

### 3.1 Dosimetric comparison of the target area

The chest wall clinical target volume (CTV) volume derived from the C-TVD method was significantly larger than that from the E-TVD method (689.68 ± 175.18 vs 412.40 ± 119.51, P < 0.001). Concurrently, the C-TVD method exhibited superior target conformity (0.85 ± 0.01 vs 0.74 ± 0.03, P < 0.001). However, the 95% prescription dose coverage of the C-TVD method was significantly lower than that of the E-TVD method (98.95 ± 0.35 vs 99.25 ± 0.39, P < 0.001). No statistically significant differences were observed in HI, MU, or high-dose volume within the target between the two methods (**Table 1**).

**Table 1:** Comparison of dose, conformity index, and homogeneity index of the target volume for conventional and ESTRO-ACROP target volume delineation (n=22).

Parameters	C-TVD	E-TVD	P
CTV_CW volume ( $\bar{x}\pm s$, cm3)	689.68 ± 175.18	412.40 ± 119.51	<0.001
V108% [M (IQR), cm3]	0.96 (2.28)	1.55 (2.30)	0.861
V95% ( $\bar{x}\pm s$, %)	98.95 ± 0.35	99.25 ± 0.39	<0.001
Dmax[M (IQR), Gy]	44.87 (0.33)	44.85 (0.46)	0.503
MU[M (IQR)]	1023.95 (197.15)	1001.33 (67.79)	0.874
CI ( $\bar{x}\pm s$)	0.85 ± 0.01	0.74 ± 0.03	<0.001
HI ( $\bar{x}\pm s$)	1.07 ± 0.01	1.07 ± 0.01	0.840

### 3.2 Dosimetric comparison for OARs

A dosimetric analysis of OARs revealed that the E-TVD method resulted in significantly lower V_20_ and D_mean_ values for the ipsilateral lung compared to the C-TVD method (both P < 0.05). No statistically significant differences were observed in dose metrics for the contralateral lung or total lung between the two methods. Concurrently, the E-TVD method yielded significantly lower values for cardiac V_20_, LAD D_max_, and LAD D_mean_ compared to the C-TVD method (all P < 0.05). No statistically significant differences were detected in cardiac V_10_, V_5_, or global cardiac D_mean_ between the two methods. Additionally, the E-TVD method was associated with a lower contralateral breast dose (P = 0.047), whereas no significant differences were observed in thyroid or liver doses (**Table 2**).

**Table 2:** Comparison of organ doses for at-risk organs between conventional target volume delineation and ESTRO-ACROP target volume delineation (n=22).

Parameters	C-TVD	E-TVD	P
Ipsilateral lung			
V20[M(IQR), %]	13.65 (1.25)	13.32 (1.59)	0.046
V10 ( $\bar{\text{x}}\pm \text{s}$, %)	24.41 ± 2.85	24.54 ± 3.29	0.524
V5 ( $\bar{\text{x}}\pm \text{s}$, %)	38.74 ± 3.45	38.88 ± 3.92	0.157
Dmean ( $\bar{x}\pm s$, Gy)	8.41 ± 0.86	8.23 ± 0.95	0.006
Contralateral lung			
V5 ( $\bar{\text{x}}\pm \text{s}$, %)	10.19 ± 3.41	10.69 ± 3.12	0.357
Dmean ( $\bar{x}\pm s$, Gy)	2.86 ± 0.37	2.74 ± 0.30	0.063
Bilateral lungs			
V20( $\bar{x}\pm s$, %)	7.27 ± 1.38	7.09 ± 1.43	0.165
V10 ( $\bar{x}\pm s$, %)	12.76 ± 1.95	12.82 ± 1.93	0.615
V5( $\bar{x}\pm s$, %)	25.02 ± 3.17	24.51 ± 3.10	0.350
Dmean( $\bar{x}\pm s$, Gy)	5.65 ± 0.55	5.46 ± 0.66	0.077
Heart			
V20[M(IQR), %]	0.16 (1.95)	0.01 (1.55)	0.029
V10[M(IQR), %]	2.93 (3.26)	3.11 (4.87)	0.545
V5 ( $\bar{x}\pm s$, %)	17.20 ± 6.04	19.69 ± 7.11	0.109
Dmean( $\bar{x}\pm s$, Gy)	3.80 ± 0.79	3.87 ± 0.64	0.689
LAD			
Dmax[M(IQR), Gy]	31.78(31.32)	25.03 (29.48)	0.002
Dmean[M(IQR), Gy]	9.01 (10.03)	8.10 (9.12)	0.013
Contralateral breast			
Dmean[M(IQR), Gy]	4.25 (0.53)	4.09 (0.66)	0.047
Thyroid			
Dmean[M(IQR), Gy]	24.19 (2.21)	24.39 (1.76)	0.610
Liver			
V5 ( $\bar{x}\pm s$, %)	7.42 ± 5.40	8.23 ± 5.54	0.113

### 3.3 Dosimetric comparison for OARs in patients with left and right breast cancer

A stratified analysis of OARs doses was performed for left-sided and right-sided breast cancer patients separately. In left-sided breast cancer patients, the E-TVD method resulted in significantly lower values for ipsilateral lung V_20_, V_10_, and D_mean_, bilateral lung V_20_, cardiac V_20_, LAD D_max_ and D_mean_, as well as contralateral breast D_mean_ (all P < 0.05). In right-sided breast cancer patients, the E-TVD method was associated with significantly lower ipsilateral lung D_mean_ and contralateral breast D_mean_ (both P < 0.05), with no statistically significant differences observed in other OAR metrics (**Tables 3**, **4**).

**Table 3:** Comparison of organs at risk doses between patients with left breast cancer (n=12).

Parameters	C-TVD	E-TVD	P
CI ( $\bar{x}\pm s$)	0.85 ± 0.01	0.75 ± 0.03	<0.001
HI [M(IQR)]	1.07 (0.02)	1.07 (0.02)	0.793
Ipsilateral lung			
V20 ( $\bar{\text{x}}\pm \text{s}$, %)	14.53 ± 2.15	13.77 ± 2.45	<0.001
V10 ( $\bar{\text{x}}\pm \text{s}$, %)	24.18 ± 2.63	23.96 ± 3.64	<0.001
V5 ( $\bar{\text{x}}\pm \text{s}$, %)	39.08 ± 3.94	37.63 ± 4.38	0.111
Dmean ( $\bar{x}\pm s$, Gy)	8.58 ± 0.94	8.12 ± 0.91	0.016
Contralateral lung			
V5( $\bar{\text{x}}\pm \text{s}$, %)	10.54 ± 2.77	11.34 ± 2.55	0.050
Dmean ( $\bar{x}\pm s$, Gy)	2.98 ± 0.30	2.80 ± 0.25	0.006
Bilateral lungs			
V20 ( $\bar{\text{x}}\pm \text{s}$, %)	6.75 ± 1.02	6.62 ± 1.16	<0.001
V10 ( $\bar{x}\pm s$, %)	12.12 ± 1.80	11.99 ± 1.55	0.071
V5 ( $\bar{x}\pm s$, %)	24.04 ± 2.98	25.55 ± 2.69	0.091
Dmean ( $\bar{x}\pm s$, Gy)	5.48 ± 0.49	5.31 ± 0.59	0.055
Heart			
V20( $\bar{x}\pm s$, %)	1.98 ± 1.89	1.69 ± 1.63	<0.001
V10 [M(IQR), %]	4.21 (5.79)	3.91 (5.83)	0.258
V5 ( $\bar{x}\pm s$, %)	18.12 ± 4.29	20.40 ± 4.73	0.362
Dmean ( $\bar{x}\pm s$, Gy)	3.98 ± 0.67	4.10 ± 0.48	0.113
LAD			
Dmax [M(IQR), Gy]	37.66 (5.56)	35.55 (6.49)	0.007
Dmean ( $\bar{x}\pm s$, Gy)	14.61 ± 5.30	13.23 ± 5.17	<0.001
Contralateral breast			
Dmean ( $\bar{x}\pm s$, Gy)	4.29 ± 0.44	4.19 ± 0.62	0.012
Thyroid			
Dmean [M(IQR), Gy]	24.79 (1.25)	24.60 (3.00)	0.470
Liver			
V5 ( $\bar{x}\pm s$, %)	6.11 ± 5.12	6.59 ± 5.08	0.071

**Table 4:** Comparison of organs at risk doses between patients with right breast cancer (n=10).

Parameters	C-TVD	E-TVD	P
CI ( $\bar{x}\pm s$)	0.85 ± 0.01	0.73 ± 0.02	<0.001
HI[M (IQR)]	1.07 (0.01)	1.07 (0.01)	0.500
Ipsilateral lung			
V20 ( $\bar{\text{x}}\pm \text{s}$, %)	14.69 ± 2.50	13.59 ± 2.76	0.173
V10 ( $\bar{\text{x}}\pm \text{s}$, %)	24.68 ± 2.78	24.22 ± 2.94	0.415
V5 ( $\bar{\text{x}}\pm \text{s}$, %)	39.66 ± 3.37	38.67 ± 3.14	0.432
Dmean[M (IQR), Gy]	8.28 (1.14)	8.05 (0.71)	0.048
Contralateral lung			
V5[M (IQR), %]	9.62 (3.78)	9.67 (2.94)	0.959
Dmean ( $\bar{x}\pm s$, Gy)	2.69 ± 0.33	2.69 ± 0.35	0.972
Bilateral lungs			
V20[M (IQR), %]	7.47 (1.40)	7.22 (1.02)	0.770
V10 ( $\bar{x}\pm s$, %)	13.91 ± 1.69	13.51 ± 1.60	0.279
V5 ( $\bar{x}\pm s$, %)	26.18 ± 3.13	25.62 ± 3.30	0.435
Dmean[M (IQR), Gy]	5.70 (0.70)	5.64 (0.84)	0.137
Heart			
V20[M (IQR), %]	–	–	–
V10 [M (IQR), %]	0.73 (2.55)	0.38 (2.50)	0.844
V5 ( $\bar{x}\pm s$, %)	16.09 ± 7.77	18.84 ± 9.44	0.354
Dmean[M (IQR), Gy]	3.53 (0.95)	3.59 (0.74)	0.695
LAD			
Dmax[M (IQR), Gy]	7.17 (5.35)	7.00 (2.59)	0.131
Dmean[M (IQR), Gy]	4.42 (1.90)	4.77 (0.96)	0.375
Contralateral breast			
Dmean [M (IQR), Gy]	4.26 (0.51)	4.02 (0.54)	0.029
Thyroid			
Dmean[M (IQR), Gy]	24.82 (3.09)	24.21 (2.50)	0.160
Liver			
V5[M (IQR), %]	10.73 (7.38)	12.21 (7.17)	0.055

## 4 Discussion

Following the publication of the ESTRO-ACROP guidelines, more explicit recommendations for target volume delineation have been established for patients undergoing implant-based breast reconstruction after breast cancer surgery. Concurrently, hypofractionated radiotherapy has increasingly emerged as the standard dose regimen for postoperative radiotherapy in breast cancer. Our study demonstrated that, in patients with implant-based breast reconstruction, the updated target volume delineation strategy confers superior dosimetric benefits for the heart, lungs, and contralateral breast during hypofractionated radiotherapy—with these advantages being more pronounced in left-sided breast cancer patients.

Conventionally, it has been posited that the complexity of target volumes in patients following implant-based breast reconstruction increases the technical difficulty of radiotherapy plan implementation. A prior study demonstrated that conventional tangential field conformal radiotherapy resulted in varying degrees of compromise in treatment plans for over half of the patients, with suboptimal target volume dose coverage and inadequate OARs protection (14). With the widespread adoption of intensity-modulated radiotherapy (IMRT), emerging evidence suggests that it is the radiotherapy technique—not the reconstruction procedure itself—that dictates the quality of radiotherapy plans (15, 16). Compared to VMAT, IMRT is associated with longer treatment times and often inferior dose conformity and homogeneity. Furthermore, shorter treatment times are known to improve compliance with DIBH techniques, which are particularly important for left-sided breast cancer. Therefore, we uniformly employed VMAT for all plans in this study. In the present study, DIBH was employed for positioning left-sided breast cancer patients, whereas free-breathing positioning was utilized for their right-sided counterparts. This discrepancy stems from the standard clinical optimization strategy for cardiac protection in left-sided breast cancer patients: DIBH can significantly reduce the radiation dose delivered to the heart and coronary arteries by increasing the anatomical distance between the heart and the chest wall.

For patients following total mastectomy, hypofractionated radiotherapy has emerged as the standard of care (17). The FAST-Forward trial demonstrated that ultra-hypofractionated regimens (26 Gy in 5 fractions) were non-inferior to standard hypofractionated radiotherapy (18). The FABREC study shows, among patients with post-reconstruction breast cancer, the local control rate and distant metastasis rate associated with hypofractionated radiotherapy were comparable to those of conventional fractionation, with no statistically significant difference in overall survival (11). Additionally, hypofractionated radiotherapy is associated with shorter treatment courses and a reduced incidence of skin adverse events (19). A separate multicenter retrospective study yielded consistent findings: the incidence of breast-related complications was significantly lower with hypofractionated radiotherapy compared to conventional fractionation (18.2% vs. 44.8%, P = 0.012) (20). Furthermore, the treatment interruption rate for hypofractionated radiotherapy was 2.7%, which was significantly lower than the 7.7% observed with conventional fractionation (P = 0.03), suggesting superior tolerability and patient compliance (11). Patients in the hypofractionated radiotherapy group exhibited higher post-treatment quality of life (QoL) scores compared to those in the conventional fractionation group, with a more pronounced advantage observed among younger patients. These findings underscore the superiority of moderate hypofractionation regimens in terms of efficacy and safety for postoperative radiotherapy in patients who have undergone breast reconstruction.

Our study demonstrated that the CI of E-TVD was significantly inferior to that of C-TVD. We attribute this finding to the “Ω”-shaped target volume design inherent to the E-TVD technique. Notably, however, the increased conformality challenge associated with the E-TVD target volume led to superior coverage with the 95% prescription dose for E-TVD, as this was necessary to meet target coverage requirements. No statistically significant differences were observed between the two techniques in terms of MU and homogeneity index HI. That said, some studies have reported an increase in MU for E-TVD, which is attributed to the complexity of its target volume (21). By excluding the implant from the E-TVD target volume, the high-dose region is positioned further away from the heart and lungs, resulting in a reduction in cardiac and pulmonary radiation exposure. Conversely, due to the inherent technical properties of VMAT, no significant improvement was observed in the low-dose region. Chang KH et al. demonstrated that in postoperative radiotherapy for patients with left-sided breast cancer who had undergone breast reconstruction and required internal mammary artery (IMA) irradiation, the application of E-TVD in conjunction with VMAT resulted in a significant reduction in radiation doses to the normal heart and LAD coronary artery tissues (22). Milligan MG et al. reported that E-TVD reduced radiation doses to the LAD coronary artery and the ipsilateral lung, while cardiac dose remained comparable. However, a trend toward increased doses to the contralateral lung and spinal cord was observed (23). Collectively, these studies consistently demonstrate that E-TVD offers superior dosimetric advantages for breast cancer patients who have undergone implant-based breast reconstruction.

Skin and implant-related adverse events remain a key concern for breast cancer patients following reconstructive surgery. Existing evidence suggests that while no statistically significant difference was observed, breast complications trended toward reduction with the application of E-TVD (24). Concurrently, hypofractionated radiotherapy has also demonstrated superiority in mitigating skin reactions. Hypofractionated radiotherapy combined with E-TVD target delineation holds promise as a potential standard treatment strategy for patients undergoing implant-based breast reconstruction in the future. Whether alternative dose fractionation regimens can yield superior dosimetric benefits warrants further investigation. Whether the reduction in cardiac and pulmonary radiation exposure translates to long-term cardiopulmonary function preservation in patients, as well as its potential implications for tumor prognosis and the development of second primary malignancies, remains to be elucidated through further research with extended follow-up durations.

This study has several limitations. First, the sample size was relatively small (n = 22). While the sample size of the present study is comparable to that of previously published dosimetric comparative studies of the same type (22, 23), the limited sample size may compromise the statistical power of the analyses. Furthermore, the sample size for the subgroup analysis was even smaller, and thus its findings should be interpreted as exploratory in nature. The stability and generalizability of these results warrant validation in future studies with larger cohorts. Notwithstanding these limitations, the present study employed a rigorous dosimetric comparative design—incorporating standardized treatment planning criteria, direct paired comparisons of the two target delineation approaches on identical image datasets, and the aforementioned target delineation consistency controls—to maximize confounding factor mitigation and enhance the internal validity and reliability of the comparative findings in the context of a small sample size. The preliminary trend of dosimetric advantages associated with E-TVD in critical organs at risk (e.g., heart, ipsilateral lung) observed in the present study aligns with the conclusions of multiple recent investigations, which lends partial support to the plausibility of our findings. Future studies should employ multi-center, large-sample prospective designs to validate the findings of the present study and assess the practical implications of these dosimetric discrepancies on long-term clinical outcomes, including cardiac toxicity, pulmonary injury, implant-related complications, and tumor control rates.

## 5 Conclusion

In conclusion, the findings of the present study align with those reported in the existing literature. The updated ESTRO ACROP target volume delineation guidelines demonstrate advantages in reducing radiation exposure to OARs among patients with left-sided or right-sided breast cancer. The long-term disease prognosis and associated benefits to normal tissues warrant further comparative investigations.

## Data Availability

The raw data supporting the conclusions of this article will be made available by the authors, without undue reservation.
